# Kinetic Compatibility as a Predictor of PTP1B Inhibitor Combination Outcomes: An Integrated Experimental and Computational Study

**DOI:** 10.1002/cmdc.70446

**Published:** 2026-08-19

**Authors:** Jonathan Trapala, Laura I. Álvarez‐Añorve, Nathaly Vasquez‐Martínez, Erika Chavira‐Suárez, Luz Vásquez‐Bochm, Deyamira Matuz‐Mares, Francisco Cortés‐Benítez, Martin González‐Andrade

**Affiliations:** ^1^ Departamento de Bioquímica Facultad de Medicina Universidad Nacional Autónoma de México Ciudad de México México; ^2^ Laboratorio de Síntesis y Aislamiento de Sustancias Bioactivas Departamento de Sistemas Biológicos División de Ciencias Biológicas y de la Salud Universidad Autónoma Metropolitana ‐ Unidad Xochimilco Ciudad de México México

**Keywords:** enzyme kinetics, molecular docking, molecular dynamics, PTP1B inhibition, type 2 diabetes mellitus

## Abstract

Although chlorogenic acid, suramin, ursolic acid, and the glycyrrhetinic acid derivative FC122 are individually known PTP1B inhibitors, no study has systematically characterized how mechanistic diversity determines the pharmacological outcome of their combinations. Here, we integrate experimental enzyme kinetics, molecular docking, molecular dynamics (MD), and membrane permeability simulations to address this question. Individual inhibitory potencies ranked SUR > FC122 > UA > CGA (IC_50_: 1.82, 3.27, 6.12, and 252 µM, respectively). Fixed‐ratio combination experiments showed two distinct profiles: additivity for the uncompetitive + mixed pair (FC122 + UA) and competitive + competitive pairs (SUR + CGA), and antagonism for the competitive + uncompetitive combination (SUR + FC122). Docking and MD provided the structural basis for these results and suggested that each combination imposes a distinct structural flexibility profile on the disordered C‐terminal region of PTP1B (residues 300–400). For the first time, the MD‐AMBER‐Umbrella‐COM protocol was applied to generate a comparative membrane permeability profile for mechanistically diverse PTP1B inhibitors, proposing a permeability order (SUR > FC122 > CGA ≈ UA). These results suggest that kinetic compatibility is a more reliable predictor of promising inhibitor combinations than binding‐site geography alone, and they offer a rational framework for designing multisite PTP1B inhibition strategies in type 2 diabetes mellitus.

## Graphical abstract

Combining PTP1B inhibitors of distinct kinetic mechanisms determines their pharmacological outcome. Integrating enzyme kinetics, docking, molecular dynamics, and membrane‐permeability simulations, we show that mechanistically compatible pairs (competitive + competitive; uncompetitive + mixed) yield additivity, whereas competitive + uncompetitive pairing produces antagonism. Kinetic compatibility predicts favorable combinations better than binding‐site geography alone, offering a rational framework for multisite PTP1B inhibition strategies in type 2 diabetes.

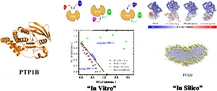

## 1 Introduction

Diabetes is a group of chronic noncommunicable metabolic diseases characterized by persistent hyperglycemia resulting from impaired insulin secretion, insulin action, or both, leading to complications affecting the kidneys, cardiovascular system, eyes, and other organs [1]. Type 2 diabetes mellitus (T2DM) represents approximately 90% of all cases globally [2, 3, 4], with an estimated 700 million by 2045. Current therapeutic approaches, including metformin, sulfonylureas, thiazolidinediones, and GLP‐1 receptor agonists, often fail to address the fundamental mechanisms of insulin resistance, highlighting the urgent need for novel therapeutic strategies [5].

**FIGURE 1:**
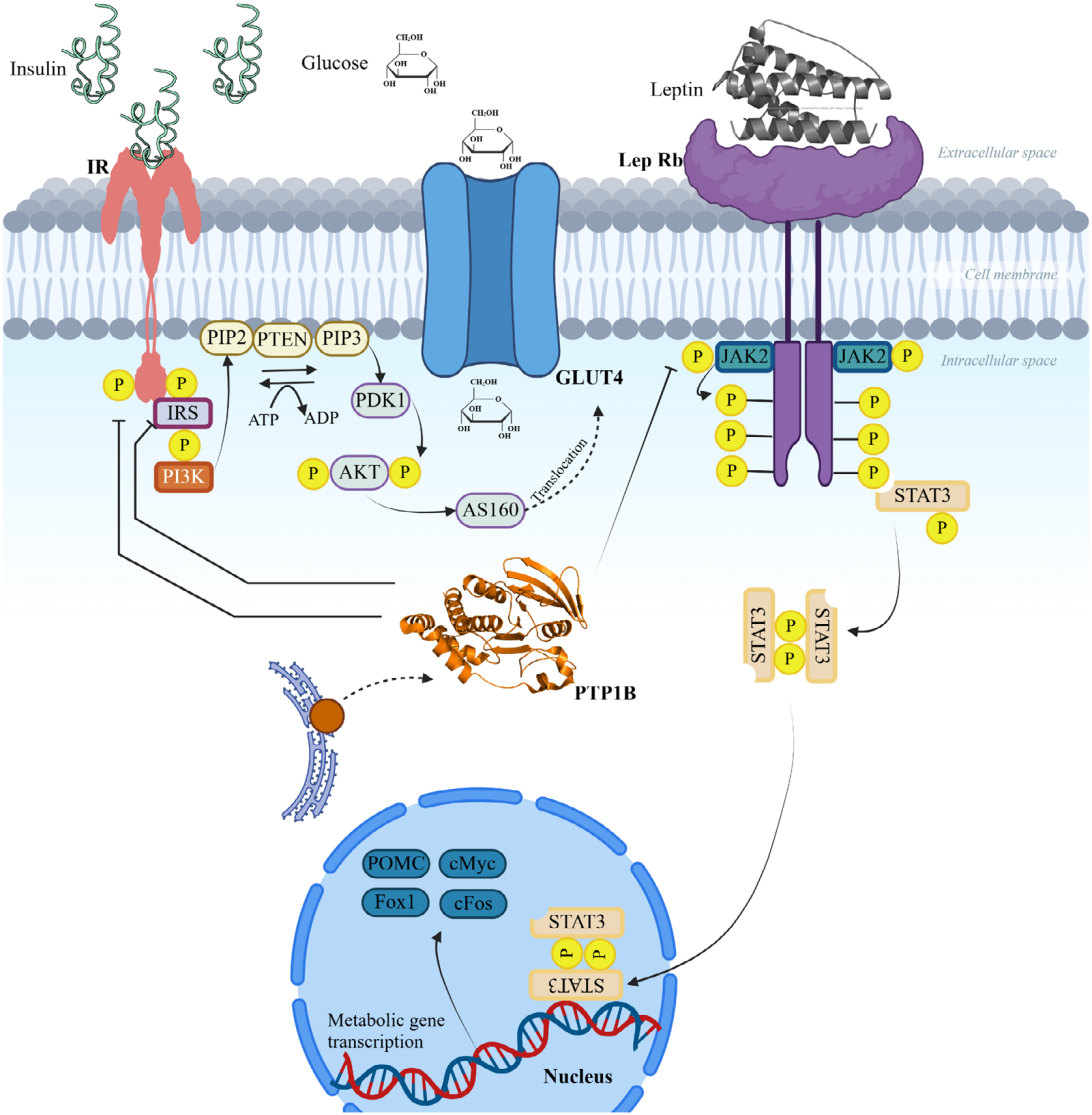
PTP1B modulates insulin and leptin signaling pathways in physiological conditions. PTP1B regulates the insulin receptor (IR) by dephosphorylating tyrosine, thereby controlling glucose‐modulated GLUT4. During leptin signaling, PTP1B regulates energy balance by dephosphorylating LepRb and JAK2, which, in turn, releases STAT3. This complex activates genes that are involved in the signaling pathway, including POMC, cMyc, and Fox1.

Protein tyrosine phosphatase 1B (PTP1B), encoded by the *PTPN1* gene, has emerged as a highly validated molecular target for the treatment of T2DM [2, 6, 7]. PTP1B is a central negative regulator of both insulin and leptin signaling that dephosphorylates specific phosphotyrosine residues on the insulin receptor and its substrates. This process reduces the translocation of glucose transporter 4 (GLUT4) and alters fatty acid synthesis (Figure 1) [7, 8, 9, 10]. Landmark genetic studies have demonstrated that PTP1B‐deficient mice exhibit enhanced insulin sensitivity, improved glycemic control, and resistance to diet‐induced obesity, establishing PTP1B as an attractive therapeutic target [11, 12].

Despite over three decades of effort, the development of clinically effective PTP1B inhibitors has faced significant challenges. These include the active site's high conservation and positive charge, poor cellular permeability, limited bioavailability, and insufficient selectivity over other phosphatases [7, 13]. Most studies have focused on characterizing each inhibitor in isolation, optimizing potency within a single mechanistic category. Four main inhibition strategies have been explored: orthosteric (active‐site‐directed), allosteric, bidentate, and gene silencing [7]. While several compounds have reached clinical trials, including ertiprotafib, trodusquemine (MSI‐1436), and antisense oligonucleotides, most have been discontinued due to insufficient efficacy, lack of specificity, or adverse effects [6, 14].

Recent advances have focused on overcoming these limitations through site‐specifically modified peptide inhibitors—particularly BimBH3 analogs with once‐weekly therapeutic potential comparable to that of semaglutide [15, 16]—allosteric inhibitors targeting the intrinsically disordered C‐terminal domain (PTP1B_301–400_) [7, 17]; and compounds that stabilize the reversibly oxidized, inactive conformation of PTP1B [18].

Importantly, inhibitors targeting distinct binding sites on PTP1B are not mutually exclusive; biophysical and molecular dynamics (MD) simulations evidence have demonstrated that molecules with distinct binding modes can simultaneously engage PTP1B [19]. This has led to the assumption that site complementarity is sufficient to predict pharmacological synergism, supported by theoretical kinetic frameworks that predict synergy when binding is non‐mutually exclusive [20]. However, whether this principle can be extrapolated to inhibitor pairs that differ in both topological binding sites and fundamental mechanistic classes remains an ongoing area of investigation. Although synergistic inhibition of PTP1B has been suggested as a strategy to enhance efficacy and reduce the required doses of individual agents, how the mechanistic class of each inhibitor determines the pharmacological nature of their combinations remains largely uncharacterized.

This work provides a systematic characterization of PTP1B inhibitor combinations selected for mechanistic diversity, using integrated experimental and computational approaches. Chlorogenic acid (CGA, competitive), suramin (SUR, competitive), ursolic acid (UA, mixed), and the glycyrrhetinic acid derivative FC122 (uncompetitive) [21] together cover the full mechanistic spectrum of reversible PTP1B inhibition modes. The central question guiding this work is not whether these compounds inhibit PTP1B—that is known—but whether binding‐site complementarity predicts synergism, additivity, or equivalence when inhibitors are combined. Docking and MD were used to validate binding‐site assignments and characterize conformational effects in the disordered C‐terminal region of PTP1B, a region increasingly recognized as pharmacologically relevant [19]. Additionally, the MD‐AMBER‐Umbrella‐COM protocol was applied to generate a comparative membrane permeability profile for these mechanistically diverse inhibitors, providing actionable information for future in vitro and in vivo studies. Understanding these findings may offer a rational basis for designing multisite combinatorial PTP1B inhibition schemes that overcome the limitations of single‐agent therapy in T2DM.

## 2 Materials and Methods

### 2.1 PTP1B Inhibition Assay

A spectrocolorimetric method previously described [22] was used to assess the inhibitory effect of the new compounds on PTP1B. Briefly, the compounds and controls were carried out in a final volume of 100 μL of 50 mM TRIS, pH 6.8, containing recombinant human protein purified (*h*PTP1B_1–400_) at 66 nM and *p*‐nitrophenyl phosphate (pNPP) at 0.5 mM. Enzyme solutions containing pNPP and inhibitors were incubated at 37 °C for 15 min. The hydrolysis product's absorbance, *p*‐nitrophenol (pNP), was measured at 405 nm using a 96‐microplate absorbance reader (Accuris SmartReader 96). The percentage inhibition was plotted as a function of inhibitor concentration. IC_50_ was calculated using regression analysis (OriginPro 2018 (64‐bit) SR1) based on Equation (1).

(1) $$\%\mathrm{PTP}1B=\frac{A_{100}}{1+{\left(\frac{i}{{\mathrm{IC}}_{50}}\right)}^{s}}$$

where %PTP1B is the percentage of inhibition, *A*_100_ is the maximum inhibition, *i* is the inhibitor concentration, IC_50_ is the concentration required to inhibit the enzyme's activity by 50%, and *s* is the cooperative degree.

### 2.2 Combination Inhibition Assay

To evaluate the pharmacological interaction between inhibitors of distinct mechanistic classes, four compounds were selected because each is a well‐characterized, structurally distinct representative of one reversible kinetic class against PTP1B with prior potency data from our group and others (CGA competitive, SUR competitive, UA mixed, FC122 uncompetitive), and together they span natural products (CGA, UA), a clinically used polypharmacological agent (SUR), and a synthetic glycyrrhetinic‐acid derivative (FC122). This composition enables a mechanism‐controlled test of combination compatibility rather than a potency‐optimized screen, which is the study's explicit aim. Their wide potency range (1.82–252 µM) was an intentional feature, enabling us to test whether the interaction outcome is governed by the mechanism rather than the potency. Combinations were prepared at fixed concentration ratios derived from the individual IC_50_ of each compound. At every tested point, the molar fraction (*f*, ranging from 0 to 1) determines the concentration of each inhibitor (Equation (2)):

(2) $$C_{1}=\left(1-f\right)\times {\mathrm{IC}}_{50,1};C_{2}=f\times {\mathrm{IC}}_{50,2}$$

such that the sum of fractional inhibitory concentrations equals unity at all points (Equation (3)):

(3) $$\frac{C_{1}}{{\mathrm{IC}}_{50,1}}+\frac{C_{2}}{{\mathrm{IC}}_{50,2}}=1$$

This constant‐ratio design establishes theoretical additivity as the baseline for comparison (Table 1), where *f* = 0 corresponds to the first inhibitor alone at its IC_50_, and *f* = 1 corresponds to the second inhibitor alone at its IC_50_, and ensures that, under a purely additive interaction, the residual enzymatic activity remains constant across all combination points.

**TABLE 1:** Inhibitor concentrations are combined to achieve a specific inhibitory fraction.

Inhibitory molar fraction	Combination FC122 + UA		Combination SUR + CGA		Combination SUR + FC122	
	FC122 (μM)	UA (μM)	SUR (μM)	CGA (μM)	SUR (μM)	FC122 (μM)
0	3.270	0	1.820	0	1.820	0
0.1	2.943	0.612	1.638	25	1.638	0.327
0.2	2.616	1.224	1.456	50	1.456	0.654
0.3	2.289	1.836	1.274	75	1.274	0.981
0.4	1.962	2.448	1.092	100	1.092	1.308
0.5	1.635	3.06	0.91	125	0.91	1.635
0.6	1.308	3.672	0.728	150	0.728	1.962
0.7	0.981	4.284	0.546	175	0.546	2.289
0.8	0.654	4.896	0.364	200	0.364	2.616
0.9	0.327	5.508	0.182	225	0.182	2.943
1	0	6.120	0	250	0	3.270

Combination assays were carried out under the same conditions described for IC_50_ determination, using *h*PTP1B_1–400_ at 66 nM and pNPP at 0.5 mM in 50 mM TRIS buffer, pH 6.8, at 37 °C for 15 min. Residual enzymatic activity was measured at each combination point and expressed as absorbance at 405 nm. To evaluate the pharmacological interactions between pairs of inhibitors, normalized isobolograms were constructed based on the mutual exclusivity model proposed by Chou and Talalay [23]. The results were plotted as the fractional inhibitory concentration (FC) of inhibitor 1 against the FC of inhibitor 2, with the diagonal line (ΣFIC = 1) serving as the additive reference. Values positioned below, on, or above this line indicate synergism (ΣFIC < 1), additivity (ΣFIC = 1), or antagonism (ΣFIC > 1), respectively.

### 2.3 Structural Model of *h*PTP1B_1–400_

A structural model of the *h*PTP1B_1–400_ protein was obtained from the AlphaFold Protein Structure Database developed by DeepMind and EMBL‐EBI (https://alphafold.ebi.ac.uk/). The UniProt code P18031 corresponds to the PTPN1 gene, which encodes human PTP1B, one of the proteins predicted by AlphaFold (code: Q9PT91). The PDB file was downloaded from the following link: https://alphafold.ebi.ac.uk/entry/P18031 [24, 25]. Subsequently, this model was subjected to MD to obtain a folding of biological relevance for the intrinsically disordered region (300–400 amino acids) [26].

### 2.4 Molecular Docking

The structural model of *h*PTP1B_1–400_, obtained following MD, was used for docking analysis. Structures UA, CGA, SUR, and FC122 were constructed and minimized in Avogadro [27]. AutoDockTools 1.5.7 [28] was used to prepare the PDB files of the protein and the compounds. Polar hydrogen atoms and the Kollman united‐atom partial charges were added to the protein structures. In contrast, Gasteiger–Marsili charges and rotatable groups were automatically assigned to the ligand structures. We used AutoDock Vina for docking [28]. Because the study probes several potential binding regions (the catalytic site, allosteric surfaces, and the disordered C‐terminal region, residues 300–400), a blind‐docking strategy was employed using a large grid box (74 × 68 × 62 Å) centered on the protein centroid (*x* = 79.9, *y* = 75.8, *z* = 39.0) to encompass the entire *h*PTP1B_1–400_ surface rather than restricting the search to the catalytic pocket; and an exhaustiveness of 25. For each ligand, the optimal conformation was selected as the lowest‐binding‐energy pose within the most populated cluster that was also consistent with the experimentally determined kinetic mechanism (catalytic‐site occupancy for the competitive inhibitors SUR and CGA; the catalytic‐domain/C‐terminal interface for the uncompetitive inhibitor FC122). The best conformational states were visualized with PyMOL 2.4.0 and Maestro Visualizer v. 21.1.020298 [29].

### 2.5 Molecular Dynamics Simulation Studies

The ligand coordinates obtained from the docking study were processed using Antechamber to generate suitable topologies for the LEaP module in AmberTools25 [30, 31]. Each structure and complex was subjected to the following protocol: hydrogen and other missing atoms were added using the LEaP module with the parm99 parameter set (ff19SB), Na^+^ counterions were added to neutralize the system, and the complexes were then solvated in an octahedral box of explicit TIP3P model water molecules, localizing the box limits at 12 Å from the protein surface. MD was performed at 1 atm and 310 K, using the Berendsen barostat and thermostat to maintain the conditions, with periodic boundary conditions and particle‐mesh Ewald (PME) sums (grid spacing of 1 Å) to treat long‐range electrostatic interactions. A 10 Å cutoff was used for computing direct interactions. The SHAKE algorithm was employed to satisfy bond constraints, enabling the use of a 2 fs time step to integrate Newton's equations, as recommended in the Amber package. Amber f99SB force field parameters were used for all residues, and Gaff force field parameters were used for the ligands [32, 33, 34]. The protocol consisted of optimizing the initial structure, followed by a 50 ps heating step at 298 K, 50 ps of constant‐volume equilibration, and 500 ps of constant‐pressure equilibration. Several independent 500 ns MD simulations were performed. Frames were saved at 100 ps intervals for subsequent analysis. The CPPTRAJ tool, part of the AMBER utilities, performed all analyses [35]. RMSD calculations were performed considering C, CA, and N; the radius of gyration (*R*_g_) was calculated with all the atoms of the protein. Mobility and structural fluctuation analyses were performed using the MDLovoFit protocol [36]. Graphs were created using Origin 2018. PyMOL and VMD were used to visualize and create MD images [29, 37].

### 2.6 Center‐of‐Mass Umbrella Restraint Code to Determine the Free Energy of Transfer Profile for a Molecule Through a Lipid Bilayer (Membrane)

The permeability of the compounds across a biological membrane was assessed by calculating the permeation free energy (PFE) using the Umbrella Sampling method within the AMBER software package. A lipid bilayer model mimicking an endothelial membrane was constructed based on the reported molar ratios. For this purpose, 2‐dioleoyl‐sn‐glycero‐3‐phosphocholine (DOPC), dioleoylphosphatidylethanolamine (DOPE), palmitoylsphingomyelin (PSM), and 1,2‐dioleoyl‐sn‐glycero‐3‐phospho‐L‐serine (DOPS) were assembled in a 5:3:1:1 molar ratio using PACKMOL‐Memgen [38, 39, 40]. The initial system setup involved positioning each parameterized ligand at the bilayer lipidic center (*z* ~ 0 Å) and generating its topology and coordinate files. The system was simulated using TIP3PBOX water (5 Å radius) and neutralized by adding counterions. The lipids were modeled using the Lipid21 force field [41]. Following energy minimization, the systems underwent a progressive heating phase (0–100 K over 5 ps) and were then heated to 310.15 K over 100 ps. A 100  ps constant‐volume equilibration followed. Steered MD were then performed to move each ligand along the *z*‐axis of the bilayer, from its initial position (*z* = 0 Å) to a final position (*z* = 32 Å), by applying a stepwise external force to its center of mass. From these trajectories, 32 initial configurations (windows) were extracted along the reaction coordinate, with approximately 1 Å separation between the centers of adjacent windows. Subsequently, MD was performed for each window over 2 ns at 310.15 K, with harmonic restraints using a force constant of 2.5 kcal/mol/Å^2^. Temperature control was achieved using the Langevin algorithm, while pressure control was performed using the Berendsen barostat. Periodic boundary conditions and the particle‐mesh Ewald (PME) method were used to treat long‐range electrostatic interactions, with a cutoff distance of 10.0 Å. The SHAKE algorithm was used to constrain bonds involving hydrogen atoms. After completing all MD windows, the PFE (kcal/mol) was reconstructed from these distributions using the Weighted Histogram Analysis Method (WHAM) [42]. All calculations were performed using a graphics processing unit (GPU, NVIDIA GeForce RTX 5090) accelerated MD engine in AMBER (pmemd.cuda), a program package that runs entirely on CUDA‐enabled GPUs [43, 44].

## 3 Results and Discussion

### 3.1 Inhibitory Potency and Pairwise Combination Profiles

**FIGURE 2:**
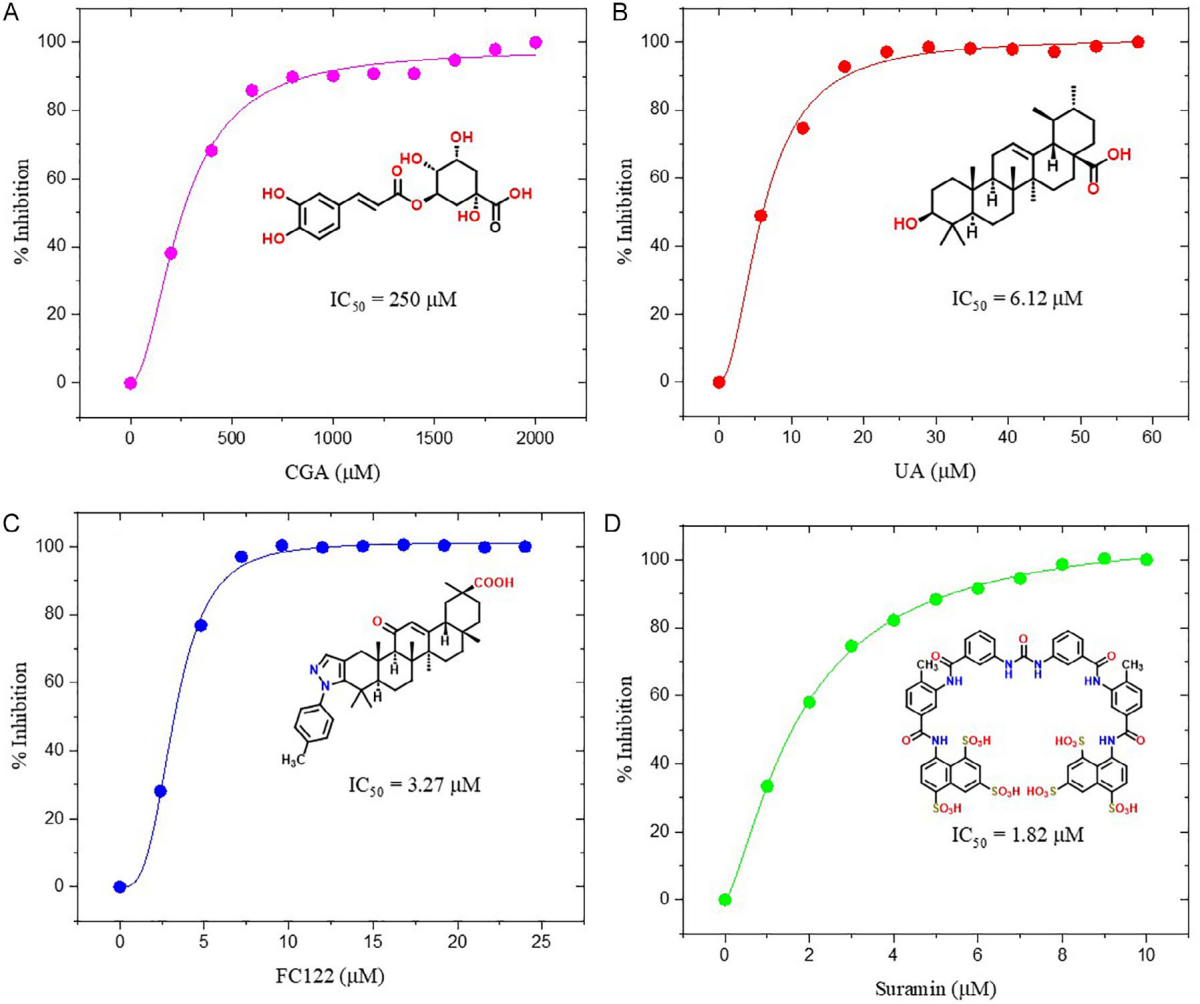
Inhibition graphs to estimate the IC_50_ of the inhibitors using *h*PTP1B_1–400_ (A) CGA, (B) UA, (C) FC122, and (D) SUR.

Before combination analysis, the inhibitory potency of each compound was determined under identical assay conditions to ensure consistency across experiments. The IC_50_ values were 252 µM for CGA, 6.12 µM for UA, 3.27 µM for FC122, and 1.82 µM for SUR (Figure 2). These values confirmed the mechanistic classification of each inhibitor and established the concentration ranges used in subsequent synergy assays [21, 22, 45].

The comparatively high IC_50_ of CGA (252 µM) is consistent with previous reports describing chlorogenic acid as a weak, reversible competitive inhibitor of PTP1B [21], and CGA was deliberately included here as the weak‐competitive extreme of the mechanistic spectrum rather than as a lead compound. Its competitive kinetic signature and concentration–response were reproducible under our assay conditions. We note, however, that at the high concentrations required to reach this IC_50_, nonspecific effects cannot be entirely excluded; CGA is, therefore, interpreted throughout as a mechanistic reference rather than as a therapeutically relevant potency.

**FIGURE 3:**
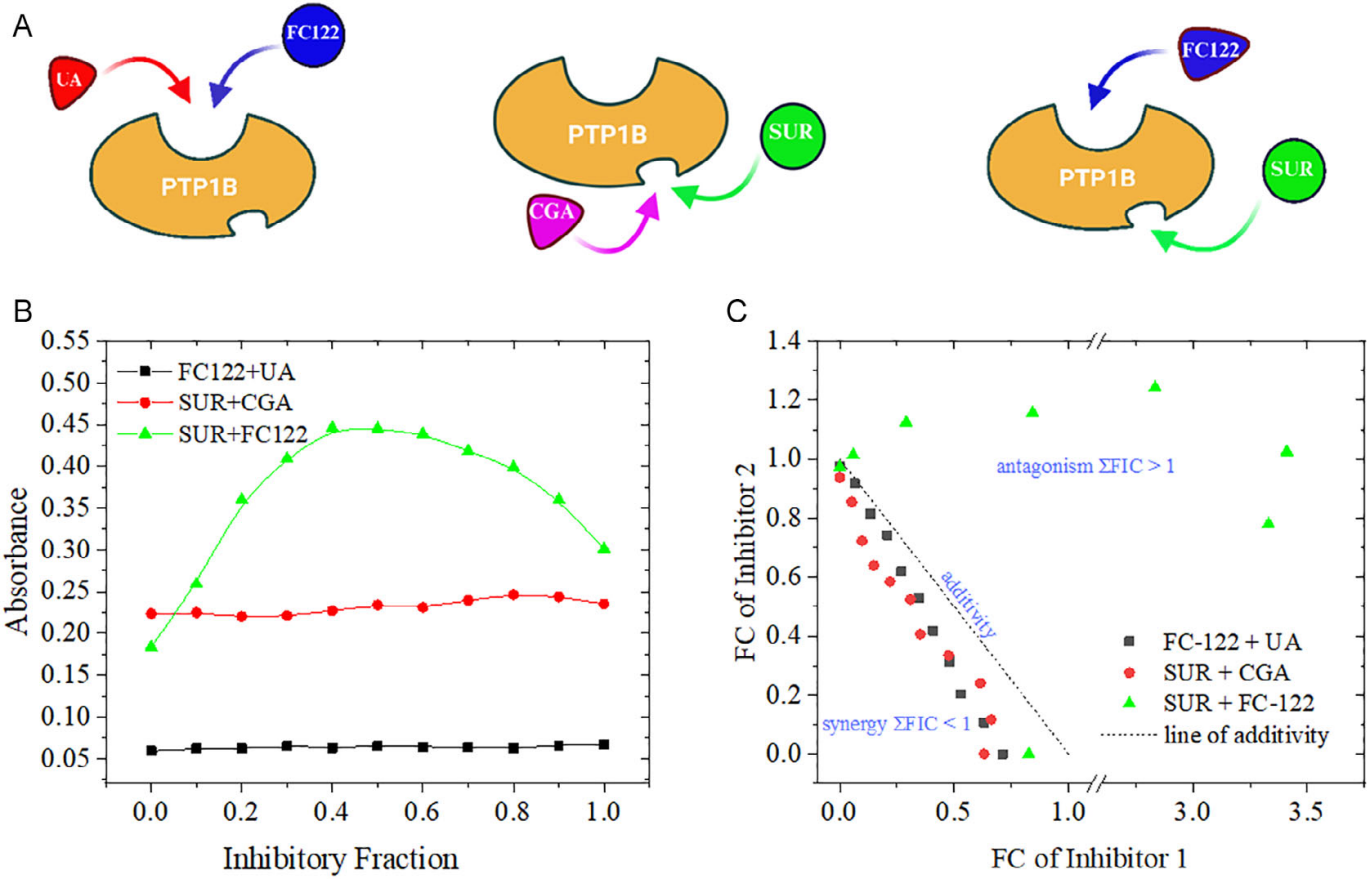
Pharmacological interaction between mechanistically distinct PTP1B inhibitors. (A) Schematic of the three binding scenarios for each inhibitor pair on *h*PTP1B_1–400_. (B) Residual enzymatic activity, expressed as absorbance at 405 nm, versus the inhibitory fraction for each combination: FC122+UA (black squares), SUR + CGA (red circles), and SUR + FC122 (green triangles). (C) Normalized isobologram of the same combinations, plotted as the fractional concentration (FC) of inhibitor 1 versus inhibitor 2; the dashed diagonal is the line of additivity (ΣFIC = 1). Points on the line indicate additivity, points below indicate synergy (ΣFIC < 1), and points above indicate antagonism (ΣFIC > 1). FC122 + UA and SUR + CGA distribute along the line of additivity, whereas SUR + FC122 lies far above it (note the broken *x*‐axis, ΣFIC; 1). Inhibitor 1 and inhibitor 2 denote, respectively, the first and second compounds named in each pair.

To assess the pharmacological interaction between inhibitors of distinct mechanistic classes, three inhibitor pairs were evaluated in combination: FC122 + UA (uncompetitive + mixed), SUR + CGA (competitive + competitive), and SUR + FC122 (competitive + uncompetitive) (Table 1). The residual enzymatic activity for each inhibitor pair is shown in Figure 3B. Because the combinations differed substantially in absolute potency, absorbance values were normalized to Abs_0_ to obtain the fraction, from which the isobologram in Figure 3C was constructed, allowing direct comparison of the pharmacological interaction independent of individual inhibitor potency.

FC122 + UA and SUR + CGA exhibited additive interactions, as evidenced by ΣFIC > 1 values approximating 1 across tested fractions, regardless of the mechanistic pairing or the 137‐fold potency disparity between SUR and CGA. In contrast, SUR + FC122 showed a distinct profile. As FC122 was progressively introduced at the expense of SUR, absorbance increased, and combination points exceeded the line of additivity, reaching ΣFIC values greater than 1 at intermediate fractions before partially recovering as the molar fraction of FC122 approached. This profile, where the combined effect is substantially weaker than predicted from individual potencies, suggests antagonism between the competitive and uncompetitive PTP1B inhibitors.

### 3.2 In Silico Characterization of PTP1B‐Inhibitor Complexes

#### 3.2.1 Molecular Docking Analysis

**FIGURE 4:**
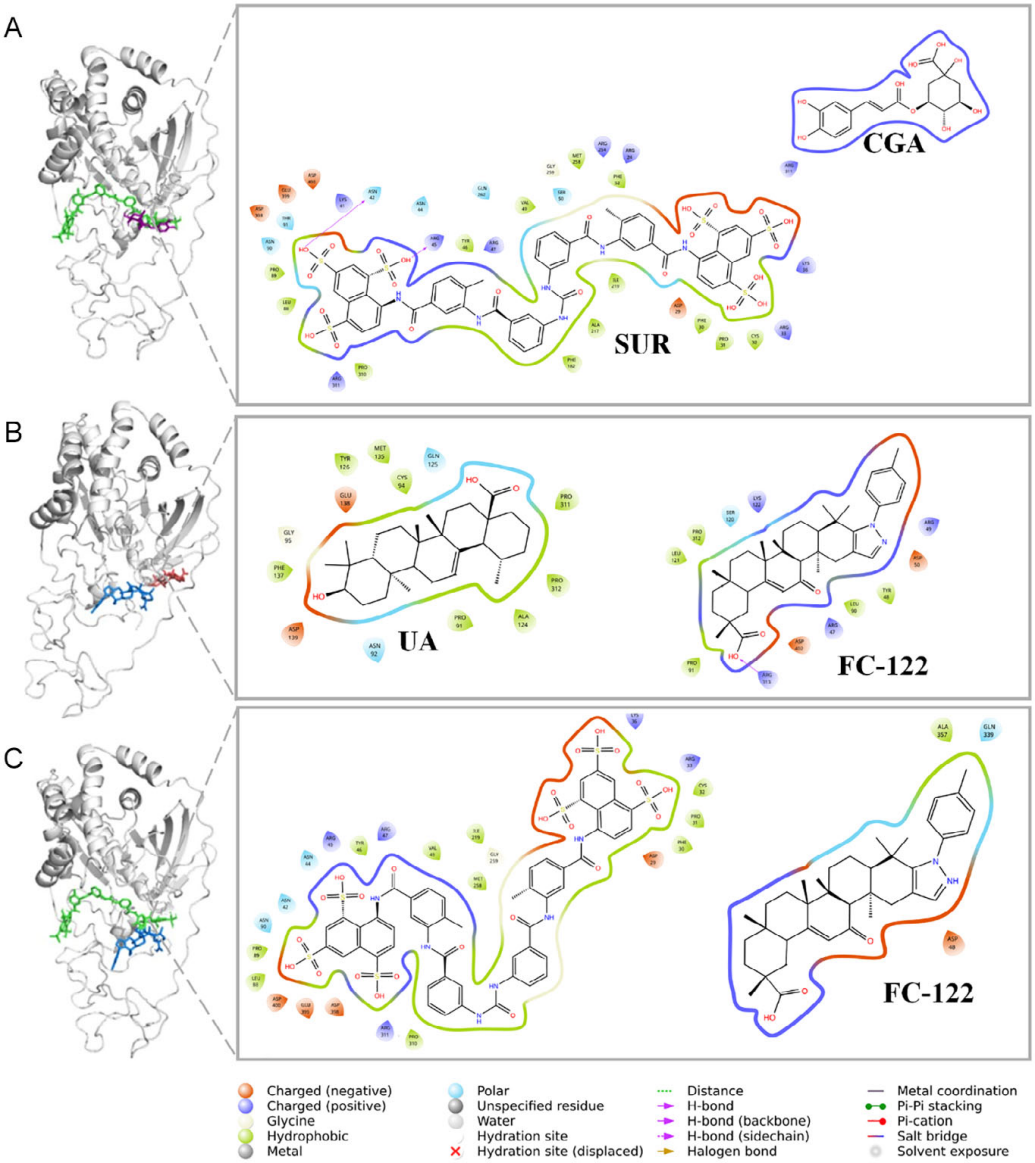
Predicted binding modes and two‐dimensional ligand–interaction maps of the inhibitors in each combination on PTP1B. In every panel, the left image shows the three‐dimensional pose of the inhibitor(s) (colored sticks) within the PTP1B structure (gray cartoon); the right image shows the corresponding two‐dimensional ligand–interaction diagram(s), in which each residue lying within the interaction cutoff of the ligand is drawn as a colored disc according to its physicochemical class and each contact according to its interaction type (see key at the bottom of the figure). The colored contour tracing of each ligand denotes its degree of solvent exposure. (A) SUR + CGA, (B) FC122 + UA, (C) SUR + FC122.

To understand the structural mechanisms driving these inhibition profiles, we analyzed the binding interactions of each inhibitor in the respective docking complex using two‐dimensional interaction diagrams (Figure 4). In the PTP1B‐SUR–CGA complex, SUR established an extensive network of interactions across the catalytic site, the Q‐loop (Arg24, Arg254, Gln262, Met258, Ile219, Phe182, and Ala217), the active site periphery, and the disordered C‐terminal region (Pro310, Arg311, Asp398, Glu399, and Asp400). The complex engaged numerous polar and charged residues through side‐chain hydrogen bonds and salt bridges via its naphthalene‐trisulfonate arms. In contrast, CGA remained largely solvent‐exposed at the periphery, forming only weak contacts (Ser118, Lys116, and Asp48) without establishing direct stabilizing interactions. These results suggest that SUR dominates the binding landscape within the complex, thereby limiting CGA anchoring potential. Nevertheless, as a competitive inhibitor, CGA can bind the free enzyme independently. This mutually exclusive occupancy of the active site suggests a simple site‐competition mechanism, consistent with the additive profile observed experimentally.

In the PTP1B‐FC122‐UA complex, both inhibitors established well‐defined and spatially distinct interaction networks. FC122 was primarily anchored at the interface between the catalytic domain and the disordered C‐terminal region (Arg311, Pro310, Arg45, Arg47, Asp48, Leu88, Tyr46, Pro89, Asp400, Leu119, Ser118, and Lys120, Arg311), forming a side‐chain hydrogen bond to Arg313 through its carboxylate, complemented by hydrophobic and charged contacts. UA occupied a distinct hydrophobic pocket flanked by acidic residues at the same interface (Phe135, Met133, Tyr124, Cys92, Gly93, Pro89, Asn90, Pro309, Pro310, Ala122, Gln123, Asp137, and Glu136). The two inhibitors interacted with a single residue (Pro310), ruling out steric interference and supporting the hypothesis that they can simultaneously occupy spatially compatible but non‐overlapping positions on PTP1B. This structural finding provides the molecular basis for the experimentally observed additive profile.

In the PTP1B‐FC122‐SUR complex, SUR again established an extensive interaction network across the catalytic site (Arg24, Arg254, Gln262, Met258, Ile219, Phe182, Ala217) and the disordered C‐terminal region (Pro310, Arg311, Asp398, Glu399, Asp400), as described above. FC122, on the other hand, established only weak interactions without forming stabilizing interactions (Ile316, Arg311, Gln339, Ala357, and Pro358). This structural displacement of FC122 is like that observed for CGA in the SUR–CGA complex, suggesting that SUR consistently dominates the binding landscape when paired with either inhibitor. However, the consequences differ; whereas CGA can still bind the free enzyme competitively when SUR dissociates, FC122 requires the enzyme‐substrate (ES) complex for binding. The depletion of the ES complex by SUR effectively limits FC122 binding, leading to the observed antagonism at intermediate molar fractions.

Collectively, the interaction profiles described above suggest qualitatively distinct binding modes that correlate with the experimental combination outcomes.

#### 3.2.2 Binding Energies and Molecular Interactions

To quantitatively support the prior observations, we next compared the theoretical binding affinities derived from docking and MD simulations with the experimentally determined inhibitory potencies.

Table 2 summarizes the theoretical binding free energies (Δ*G*_Theoretical_) calculated from docking and MD for the four PTP1B‐ligand complexes. A more negative Δ*G*_Theoretical_ indicates a more favorable interaction and, therefore, a higher binding affinity. Across both approaches, the affinity ranking followed the same order: SUR < FC122  <  UA < CGA, with docking values of −25.37, −10.16, −8.62, and −8.05 kcal/mol, and MD‐derived values of −48.12, −43.38, −41.90, and −20.57 kcal/mol, respectively. Notably, compounds with more negative Δ*G*_Theoretical_ values exhibited lower IC_50_ (SUR < FC122  <  UA < CGA: 1.82, 3.27, 6.12, and 252 μM, respectively), confirming that computational binding affinity rankings agree with experimental inhibitory potency.

**TABLE 2:** IC_50_ and Δ*G*_T_ from docking and MD studies of PTP1B‐inhibitor complexes.

Complex	**CGA‐SUR**		**FC122–SUR**		**UA–FC122**	
	CGA	SUR	FC‐122	SUR	UA	FC‐122
IC_50_ (μM)	252.38 ± 12.27	1.82 ± 0.004	3.27 ± 0.06	1.82 ± 0.004	6.12 ± 0.26	3.27 ± 0.06
Docking Δ*G*_T_ (kcal/mol)	−8.05	−28.63	−9.24	−25.37	−8.62	−10.16
MD Δ*G*_T_ (kcal/mol)	−36.6952	−41.1323	−41.6752	−21.0609	−29.2487	−28.3773

Abbreviations: Δ*G*_T_, theoretical total binding free energy; CGA, chlorogenic acid; FC‐122, glycyrrhetinic acid derivative; MD, molecular dynamics simulation; SUR, suramin; UA, ursolic acid.

#### 3.2.3 Conformational Stability Analysis

**FIGURE 5:**
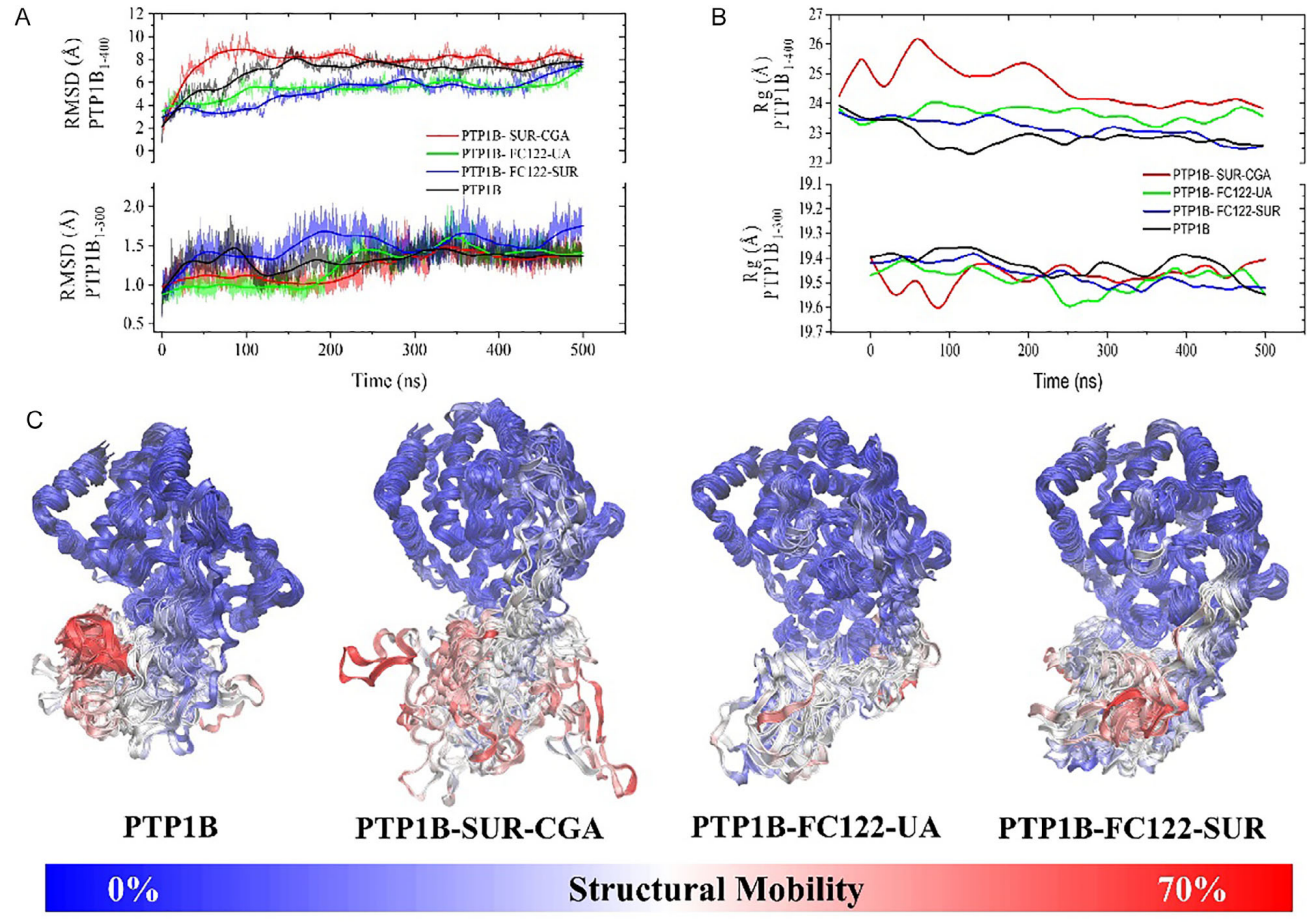
Conformational stability of PTP1B and its inhibitor complexes over 500 ns of molecular dynamics. (A) Backbone RMSD as a function of time for the full‐length protein (*h*PTP1B_1–400_, upper panel) and for the catalytic domain alone (*h*PTP1B_1–300_, lower panel), for the apo protein (black) and the three complexes: PTP1B–SUR–CGA (red), PTP1B–FC122–UA (green), and PTP1B–FC122–SUR (blue). The catalytic domain remains stable (≈1–2 Å), whereas the RMSD of the full‐length protein is dominated by the intrinsically disordered C‐terminal region (residues 301–400). (B) Corresponding radius of gyration (R*g*) for *h*PTP1B_1–400_ (upper) and *h*PTP1B_1–300_ (lower). (C) Per‐residue structural mobility mapped onto representative superimposed structures of the apo protein and the three complexes, colored from low (0%, blue) to high (70%, red) mobility, showing a rigid catalytic core and a highly mobile C‐terminus. In A and B, faint traces are the per‐frame values, and bold traces are the running average.

MD simulations were performed on all docking complexes to evaluate structural stability and conformational flexibility. RMSD values provide a quantitative measure of structural deviation from the initial conformation, indicating the system's overall stability during the simulated trajectory. For our systems, the RMSD showed progressive stabilization after 300 ns of simulation. The trajectories show that the catalytic domain (residues 1–300) is well equilibrated—its RMSD plateaus at ≈1–2 Å (Figure 5A, lower panel) and its radius of gyration remains essentially constant at ≈19.4 Å (Figure 5B, lower panel) throughout the trajectory—whereas the larger global fluctuations of the full‐length protein (*h*PTP1B_1–400_) arise from the intrinsically disordered C‐terminal region (residues 301–400). Over the final 50 ns, the RMSD to 1.4–1.7 Å with a standard deviation ≤0.14 Å for the catalytic domain (residues 1–300) and 9.6–14.6 Å for the full‐length protein with a standard deviation ≤0.94 Å (residues 1–400), confirming that the elevated global deviation originates in the disordered C‐terminal region rather than in the catalytic core (Figure 5A). The *R*_g_ showed distinct compaction profiles for each system. The PTP1B‐FC122‐UA complex maintained a consistently compact conformation, like the free protein. In contrast, the PTP1B‐SUR–CGA complex showed marked expansion, while PTP1B‐FC122‐SUR exhibited the opposite trend, revealing a potentially more rigid global conformation (Figure 5B). These differences suggest that the nature of the inhibitor combination, rather than the presence of individual ligands, determines the global architecture of the enzyme.

Structural mobility characterization using MDLovofit complemented these analyses by providing a visual description of the molecular motion patterns of PTP1B. The catalytic region (1–300 amino acids) is the most immobile across all complexes, as expected, and the unstructured region is the most structurally mobile (Figure 5C). Importantly, the degree of conformational restraint imposed on this region was highly specific to each inhibitor combination. The pairing of uncompetitive and mixed inhibitors (PTP1B‐FC122‐UA complex) appears to produce the greatest reduction in the structural flexibility of this region; the uncompetitive‐competitive combination (PTP1B‐FC122‐SUR complex) appears to yield intermediate stabilization; and the dual competitive inhibitors (PTP1B‐SUR–CGA complex) appear to result in the least structural constraint.

Taken together, these dynamic profiles are consistent with the binding‐site complementarity identified by docking and with the experimental outcomes. When two inhibitors compete for the same site, as in the SUR + CGA pair, mutual exclusivity limits their combined effect, with each compound contributing independently to the inhibition of free enzyme. This mutual exclusivity also explains why the 137‐fold potency difference between SUR and CGA confers no additional inhibitory benefit upon combination. Consistently, MD shows that PTP1B‐SUR‐CGA retains the highest structural mobility in the disordered C‐terminal region among the three complexes, suggesting that exclusive active‐site occupancy does not propagate conformational constraints toward the C‐terminal domain.

The additive interaction between FC122 and UA can be attributed to kinetic cooperativity in their inhibitory mechanisms. We propose a model in which UA reduces catalytic turnover, thereby prolonging the lifetime of the ES complex. This mechanism could indirectly enhance FC122 occupancy, thereby potentiating the total inhibitory effect beyond additive levels. This observation aligns with the substantial reduction in C‐terminal structural mobility observed in MD for this complex, indicating that kinetic cooperativity imposes a more pronounced conformational constraint on this domain.

The antagonism between SUR and FC122 is surprising, as these two inhibitors bind to distinct regions of the enzyme. FC122 can bind only after the substrate has entered the active site, requiring the existence of the ES complex. Conversely, SUR, as a competitive inhibitor, may block the active site before substrate entry, potentially limiting the formation of the ES complex on which FC122 depends. Under this framework, at intermediate concentrations of both inhibitors, each compound could undermine the binding competence of the other, as we observed. The MD data were consistent with this hypothesis. PTP1B‐FC122‐SUR was the most conformationally compact of the three complexes, which may reflect a protein trapped in a state where neither inhibitor can act optimally. While our data support this model, further experimental work will be necessary to fully elucidate the proposed mechanism.

The observation that the pharmacological outcome of PTP1B inhibitor combinations is governed by kinetic compatibility rather than binding‐site complementarity alone may constitute the central conceptual contribution of this work. It could provide a more reliable design rule for the development of new multi‐site PTP1B inhibitor combinations than structural criteria alone.

#### 3.2.4 Theoretical Permeation Free Energy Profile

An essential aspect of the drug discovery and development process is that many molecules that perform well in in vitro studies often fail to exhibit the expected effects when tested in vivo. This latter aspect concerns the pharmacokinetics of the molecules, primarily their distribution within complex systems until they reach their molecular targets. One of the limiting steps in adequate drug distribution is crossing cell membranes to reach the site of action. In this work, we used MD to calculate the theoretical free‐energy profile of permeation for the studied PTP1B inhibitors (CGA, UA, FC122, and SUR) using the AMBER Umbrella COM restraint code. A key aspect of this protocol was constructing a lipid bilayer of human endothelial cells to maximize physiological relevance.

**FIGURE 6:**
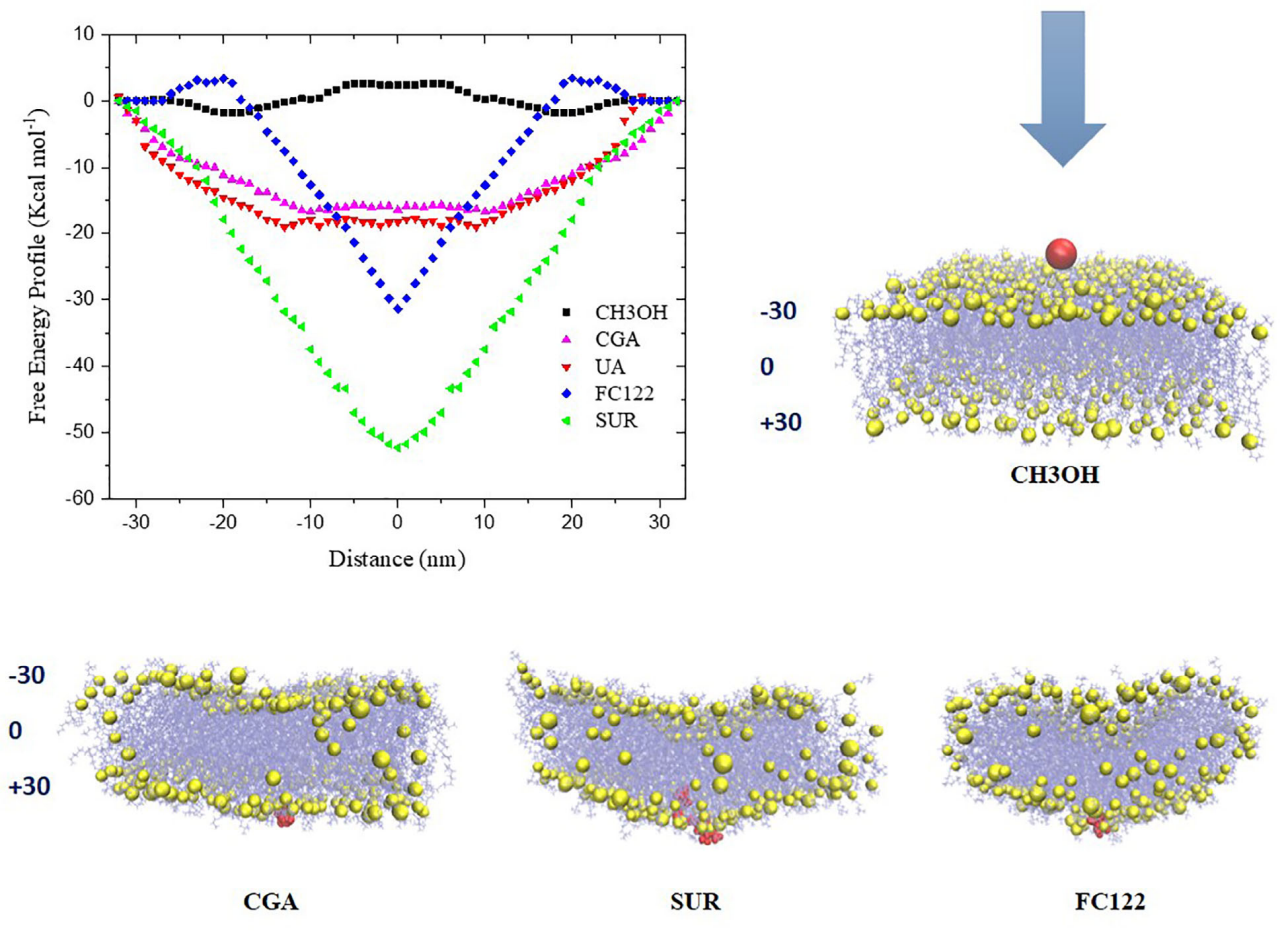
Pushing of inhibitors across the *z*‐axis of a lipid bilayer. The plot shows the free energy profile for the movement of an inhibitor and a control molecule (CH_3_OH) along the *z*‐axis of an endothelial bilayer. The distance on the *x*‐axis represents the position relative to the membrane, where −30 and +30 nm represent the aqueous phase surrounding the upper and lower surfaces of the bilayer, and 0 represents the center of the membrane. The 3D models of the inhibitors crossing the lipid bilayer were constructed from the final frame of the MD data using VMD.

The free energy profiles revealed distinct permeation behaviors among the four inhibitors (Figure 6). CGA and UA exhibit minimum energetic barriers of approximately −15 kcal/mol. This suggests that they have comparable theoretical permeabilities and similar structural or physicochemical features that facilitate penetrability into the specific lipid environment and translocation across the lipid bilayer. The inhibitor FC122, with a minimum energy barrier of −31 kcal/mol, exhibits higher theoretical permeability, doubling the energetic stabilization observed by CGA and UA. This energetic increase may be attributed to more favorable interactions with the membrane's hydrophobic components, arising from optimized lipophilic properties or a charge distribution that facilitates insertion and migration through the lipid core of the bilayer. Initially, the interaction with the polar head groups of the phospholipids appears to reduce the interaction energy (between 25 and 15 nm in the lipid bilayer). The SUR compound exhibits the highest theoretical permeability among the inhibitors, with a minimum energy barrier of approximately −52 kcal/mol. This stability is 3.7 times that of CGA and UA, and 1.8 times that of FC122 (Figure 6). This highly favorable minimum energy barrier may indicate optimal molecular properties for membrane permeability, suggesting a structure that optimizes interactions with the lipid environment while minimizing the entropic penalties associated with translocation. Methanol (CH_3_OH), a polar compound, was included as a control for this lipid bilayer. The minimum energy barrier was positive (+2.4 kcal/mol) as expected. This result confirms that the model correctly penalizes the permeation of hydrophilic molecules, thereby validating the simulation setup. AMBER‐Umbrella‐COM simulations suggest a permeability pattern for the inhibitors under investigation: SUR > FC122 > CGA ≈ UA. This comparative permeability profile may be valuable for optimizing pharmacokinetic properties of these inhibitors in future *“*in vivo*”* studies.

This predicted ranking is relative rather than absolute, and it is consistent with independent physicochemical and pharmacological evidence: suramin is a large, highly charged polysulfonated molecule whose well‐documented poor passive membrane permeability necessitates parenteral administration [46], whereas the relative ordering of the remaining compounds is in line with their reported lipophilicity (logP) profiles [47]. Because the umbrella‐sampling/COM protocol yields comparative rather than absolute permeabilities, direct experimental validation—for example, PAMPA and Caco‐2 permeability assays—will be required to confirm the predicted order and is proposed as the natural next step of this work.

## 4 Conclusion

This study provides a systematic, mechanism‐driven characterization of PTP1B inhibitor combinations that goes beyond the conventional single‐agent paradigm. By integrating fixed‐ratio combination kinetics with molecular docking, MD, and membrane permeability simulations, we show that kinetic compatibility—not binding‐site geography alone—governs the pharmacological outcome of PTP1B inhibitor combinations.

The uncompetitive + mixed pair (FC122 + UA) produced additive inhibition: at the tested concentrations, each compound alone already occupies the shared active‐site region near‐maximally, so the mixture reproduces rather than exceeds the effect of the more potent component. This stable co‐occupancy was structurally corroborated by the pronounced reduction in C‐terminal conformational mobility observed in MD simulations of this complex. By contrast, the competitive + competitive pair (SUR + CGA) exhibited additivity consistent with mutual exclusion at the active site, where a 137‐fold potency difference between the agents confers no synergistic benefit. Most strikingly, the competitive + uncompetitive pair (SUR + FC122) produced functional antagonism: competitive blockade of substrate entry by SUR depletes the very enzyme–substrate complex required for FC122 binding, a mechanism reflected in the anomalous compaction of the PTP1B–SUR–FC122 complex observed in simulation.

The MD‐AMBER‐Umbrella‐COM protocol applied here to a mechanistically diverse panel of PTP1B inhibitors, yielded a comparative membrane permeability hierarchy (SUR > FC122 > CGA ≈ UA) that provides actionable guidance for candidate prioritization in future “in vitro” cellular and “in vivo” studies. Notably, despite its high enzymatic potency, SUR's physicochemical profile and large molecular size raise concerns regarding selectivity and bioavailability that warrant further investigation.

Taken together, these findings offer a principled framework for the rational design of multi‐site PTP1B inhibition strategies, one in which kinetic mechanism, rather than binding‐site geography alone, serves as the primary criterion for combination selection. Concretely, such screening can be implemented in five steps: (1) determine the reversible kinetic mechanism and IC_50_ of each candidate inhibitor; (2) classify candidates by mechanism (competitive, uncompetitive, mixed, or noncompetitive); (3) flag mechanistically incompatible pairs—most importantly a competitive, substrate‐blocking inhibitor combined with an uncompetitive, ES‐complex‐dependent binder, which our data predict to be antagonistic—and prioritize compatible pairs; (4) test the prioritized pairs in fixed‐ratio combination assays analyzed by isobologram and combination‐index methods; and (5) computationally validate the retained hits with docking, MD, and the membrane‐permeability protocol described here.

Combining inhibitors that are kinetically cooperative—rather than merely topologically complementary—may help overcome the long‐standing limitations of monotherapy in T2DM, including insufficient efficacy and the redundancy of insulin signaling networks. We propose that systematic kinetic compatibility screening, integrated with the computational pipeline described here, represents a tractable and generalizable strategy for identifying favorable, non‐antagonistic combinations among the growing repertoire of PTP1B inhibitors currently under investigation.

## Conflicts of Interest

The authors declare no conflicts of interest.

## Data Availability

The data that support the findings of this study are available from the corresponding author upon reasonable request.
